# Omecamtiv Mecarbil in the treatment of heart failure: the past, the present, and the future

**DOI:** 10.3389/fcvm.2024.1337154

**Published:** 2024-03-19

**Authors:** Shujing Zhou, Ying Liu, Xufeng Huang, Chuhan Wu, Róbert Pórszász

**Affiliations:** ^1^ Department of Pharmacology and Pharmacotherapy, Faculty of Medicine, University of Debrecen, Debrecen, Hungary; ^2^ Department of Cardiology, Sixth Medical Centre, Chinese PLA General Hospital, Beijing, China; ^3^ Faculty of Dentistry, University of Debrecen, Debrecen, Hungary

**Keywords:** Omecamtiv Mecarbil, positive inotrope, calcium sensitizer, myosin activator, heart failure

## Abstract

Heart failure, a prevailing global health issue, imposes a substantial burden on both healthcare systems and patients worldwide. With an escalating prevalence of heart failure, prolonged survival rates, and an aging demographic, an increasing number of individuals are progressing to more advanced phases of this incapacitating ailment. Against this backdrop, the quest for pharmacological agents capable of addressing the diverse subtypes of heart failure becomes a paramount pursuit. From this viewpoint, the present article focuses on Omecamtiv Mecarbil (OM), an emerging chemical compound said to exert inotropic effects without altering calcium homeostasis. For the first time, as a review, the present article uniquely started from the very basic pathophysiology of heart failure, its classification, and the strategies underpinning drug design, to on-going debates of OM's underlying mechanism of action and the latest large-scale clinical trials. Furthermore, we not only saw the advantages of OM, but also exhaustively summarized the concerns in sense of its effects. These of no doubt make the present article the most systemic and informative one among the existing literature. Overall, by offering new mechanistic insights and therapeutic possibilities, OM has carved a significant niche in the treatment of heart failure, making it a compelling subject of study.

## 1 Introduction

Heart failure affects a substantial population worldwide, constituting a primary cause of mortality, hospitalization, and diminished quality of life, and imposing a substantial healthcare system burden, addressing it should be considered a global health priority (1–3). The rising prevalence of heart failure, survival rates, and demographic aging have resulted in an increasing cohort of patients progressing to advanced stages of heart failure, leading to a more sophisticated situation than ever before. The complicated scenario poses formidable challenges to clinical practitioners as such patients often manifest severe symptoms, marked deterioration in the quality of life, display attenuated responsiveness to regular therapeutic modalities, and have a bleak 5-year survival from diagnosis. In general, clinical outcomes remain less satisfying in patients not deemed suitable for protracted mechanical circulatory support or cardiac transplantation which is believed to be the only definitive resolution for this issue, yet instead of going palliative, voices calling for necessary consideration for a delimited fraction of late-stage heart failure patients for intensive therapeutic interventions still exist (4).

Within this context, the development of pharmacological agents capable of addressing various heart failure subtypes has become an imperative forefront pursuit, as it is a general belief that pharmacological intervention stands as an established and effective approach for the management of heart failure. Especially, while it may not encompass an exhaustive analysis of specific scenarios, clinical guidelines incorporate substantially both past clinical experiences and recent research findings, sharing hopes with patients and physicians constantly (5). From this end, the exploration of more novel drugs to enrich such guidelines is still meaningful. Therefore, the objective of the present article is to focus on Omecamtiv Mecarbil (OM), one of the many new inotropic agents, a rising star in the field, but also start from the pathophysiological basis of heart failure as well as its classification and the strategies of drug design, sharing a new mechanistic insight and therapeutic opportunity, providing a comprehensive summary of its progress made over the past 10 years in the field.

## 2 Heart failure: the pathomechanisms and classifications

Heart failure is defined as a pathological condition in which the heart is unable to sustain sufficient cardiac output, and thus inadequate oxygen can be transported to various organs and local tissues for their daily metabolic demands.

It is believed that a failing heart can be a result of diverse abnormalities, such as structural problems and functional changes that make it more susceptible to reduced cardiac output and elevated intracardiac filling pressures at rest or during exercise (6).

In general, heart failure can be subdivided into numerous distinct types. Principally speaking, as heart failure is attributed to impaired diastolic and systolic function directly, this can be employed as a classification scheme.

From the emergency perspective, the condition can also be categorized into 2 principal forms, chronic and acute heart failure. Chronic heart failure lasts a long time, mostly months or years, typically by the heart's ability to achieve adaptation, such as dilation and hypertrophy in response to provocative factors. Nonetheless, these adaptive reactions, if maintained constantly, are detrimental. Conversely, acute heart failure is characterized by an abrupt onset and potential immediate life-threatening consequences, as the heart is deprived of the temporal capacity for compensatory adaptation.

Nowadays, more accurate classification is made by measuring the proportion of blood pumped out during a single contraction (i.e., ejection fraction, EF), according to which, heart failure can also be divided into case series with preserved ejection fraction (HFpEF), mildly reduced ejection fraction (HFmrEF), and reduced ejection fraction (HFrEF), respectively (2).

## 3 The rationale of drug therapy in heart failure

Currently, the treatment methods for heart failure depend on two different mechanisms (7, 8). The first mechanism has been extensively explored and widely used in current heart failure management, with the focus on blocking the excessive compensation of the neurohormonal system (9). The second mechanism, however, aims to augment myocardial contractility, accomplished by either indirectly increasing the intracellular Ca2+ level, indirectly sensitizing the cellular responsiveness toward a given Ca2+ concentration, or directly influencing the interaction between myosin and actin (10). OM falls within the very last category.

Table 1 presents the currently available (except for OM which is now awaiting approval for marketing) pharmacotherapeutic options for heart failure and corresponding side-effects. Overall, OM is free of the known adverse effects of other drugs, although other concerns were proposed which will be discussed at the last part of the present article.

**Table 1:** List of different drugs in use in treating heart failure.

Drug class	Example	Adverse effects	Publication
β blockers	Metoprolol	Intermittent claudication, bradycardia	PMID: 23796325
Sinoatrial node inhibitor	Ivabradine	Luminous phenomena, bradycardia, headache	PMID: 30266358, PMID: 16214830
Loop diuretics	Furosemide	Hypokalemia	PMID: 23852396
ACE inhibitor	Ramipril	Coughing, angioneurotic edema	PMID: 12140728
ARB	Losartan	Notable differences in the efficacy against tissue pathogenesis and related clinical outcomes	PMID: 31433752, PMID: 18404673
Aldosterone antagonist	Spironolactone	Hyperkalemia	PMID: 31433752, PMID: 18404673
Na+/K+ pump inhibitor	Digitalis	Ca2+ overload	PMID: 2580875
β1 receptors activator	Dobutamine	Ca2+ overload	PMID: 10742697
PDE3 inhibitor	Milrinone	Ca2+ overload	PMID: 19060915, PMID: 3973022
Ca2+ sensitizer	Pimobendan, Levosimendan	Also Ca2+ overload, as they are not pure Ca2+ sensitizer	PMID: 16418450, PMID: 8043944, PMID: 24547784
Myosin activator	Omecamtiv Mecarbil (OM)	Only theoretical concerns/still under investigation, unvalidated	PMID: 24900233, PMID: 21544138

### 3.1 Designing the heart failure drugs: a neuro-humoral view

As we know, a failing heart is a heart that cannot adequately cope with the increased demands of maintaining sufficient cardiac output during periods of intense physical exertion. As heart failure progresses, there is a corresponding decline in resting cardiac function, which may eventually reach an irreversible state.

In less severe situations, the resting cardiac output may still exhibit normal behavior, owing to the compensatory mechanisms activated. These compensation mechanisms are achieved via the activation of neuro-humoral systems to maintain relatively normal arterial pressure. However, when the heart is no longer compensable (i.e., heart failure), such mechanisms become a huge burden to human body, and thus in need of mitigated.

From a holistic point of view, the neuro-humoral systems may be roughly categorized into the sympathetic nervous system and conduction system in the heart which collectively stand for the prefix “neuro-”, and the renin-angiotensin-aldosterone system (RAAS) which comprises the “-humoral” suffix (11). Upon a pathological insult to the myocardium, a failing heart will experience a considerable drop in mean arterial pressure (MAP) due to an ensuing reduction in cardiac output. Thereafter, neuro-humoral systems will be activated. Therefore, even more than a century ago, pioneers had been thinking of giving patient with heart failure drugs that could activate the aforementioned systems to rescue the situation.

#### 3.1.1 The basis of drug design: aiming at the “neuro-” systems

Over-activation of the sympathetic nervous system is common in heart failure, usually resulting in heightened release of catecholamines (mainly adrenaline and noradrenaline) that stimulate the adrenergic receptors, leading to increased heart rate, increased workload on the heart, and remodeling of the heart muscle, contributing to the detriment of the heart. In this context, β blockers such as bisoprolol, carvedilol, and metoprolol, significantly reducing heart rate, improving pumping efficiency, lowering blood pressure, and preventing cardiac structural changes, are representative of this class, with only a few adverse effects (i.e., intermittent claudication and bradycardia) from statistic investigations (12).

As for the conduction system, reducing heart rate has long been the therapeutic goal for heart failure. This is because the increased heart rate in heart failure patients is problematic as it increases myocardial oxygen demand and reduces myocardial perfusion. Ivabradine, the representative drug of sinoatrial pacemaker inhibitors, selectively inhibits the funny current and thus lowers the heart rate, treating heart failure (13, 14).

Together, β blockers and Ivabradine help avoid over-stimulation of the “neuro-” systems, ultimately ensuring the heart won't fall into exhaustive status when compensation is not realistic (15).

#### 3.1.2 The basis of drug design: utilizing the “-humoral” system

Given that the RAAS represents one of the principal driving forces in the pathophysiology of heart failure, its inhibition plays an important role in the neuro-humoral systems of heart failure. There are quite some players in the system, including diuretics Angiotensin-Converting Enzyme (ACE), Angiotensin II type 1 (AT-1) receptor, and aldosterone. Drugs acting aiming at them are of popular options in heart failure treatment.

Loop diuretics represented by furosemide were a mainstay of treatment since very old days. The treatment logic behind is to treat symptoms of fluid accumulation (i.e., edema), one of the major manifestations of heart failure, but regardless the broad usage, their efficacy and safety remain questionable due to the lack of evidence, letting alone the risk of hypokalemia (16).

Angiotensin, as its name implies, possesses the function of raising blood pressure which may disturb the cardiac output in heart failure, and thus its inhibitor (e.g., ramipril) can be used as a drug for alleviation. However, ACE inhibitor was found with certain side effects including coughing and angioneurotic edema. Plus, due to the existence of alternative pathways for the formation of angiotensin II, ACE inhibitors rarely rescue patient from a long-term perspective. Together, they made optimization of this pharmacotherapeutic strategy necessary (17). From this end, AT-1 receptor blocker (ARB) such as losartan came into being. The only drawback of ARB that is clear so far, is the notable differences in the efficacy against tissue pathogenesis and related clinical outcomes between each specific drug under this category (18). Therefore, more careful assessment of patient's holistic health condition should be made before prescribing such drugs.

In addition to angiotensin, downstream effectors of RAAS, particularly aldosterone, merit consideration as viable candidates for pharmacological intervention. In fact, several drugs for this purpose have been in use for a long time, exemplified by spironolactone, which, regretfully, is not devoid of imperfections and associated with a discernible risk of hyperkalemia (19, 20).

### 3.2 Designing the heart failure drugs: a positive inotropic view

**Figure 1:**
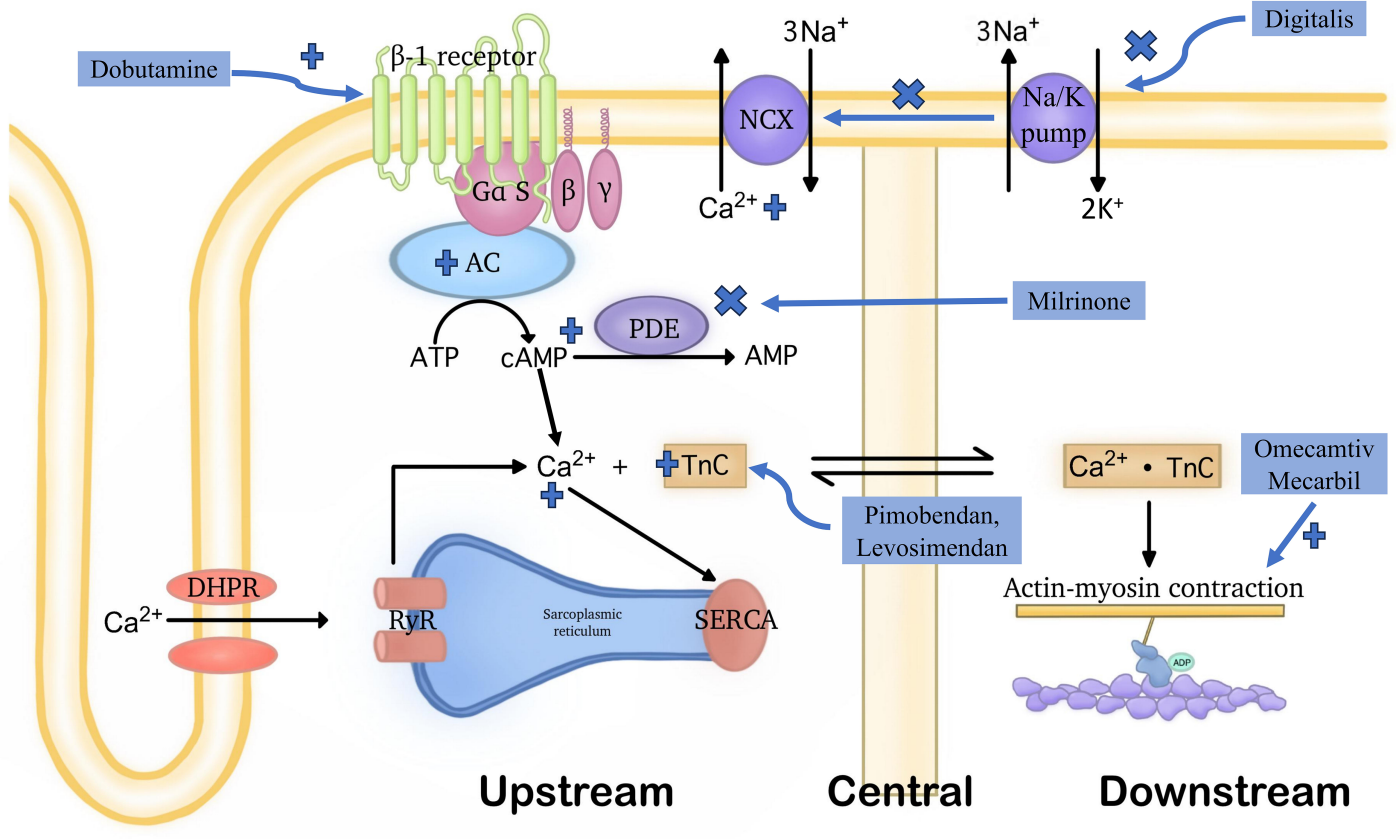
Demonstration of the general functioning mechanisms of various positive inotropic agents. Positive inotropic drugs can improve cardiac pump function through three distinct mechanisms: (1) upstream mechanism: medications acting through this mechanism influence various signaling pathways and transporter proteins to increase intracellular Ca2+ concentrations. This includes inhibiting the function of the Na+/Ca2+ exchanger located in the cell membrane, activating β1 receptors, or targeting PDE3. (2) central mechanism: drugs acting through this mechanism enhance the affinity of troponin C for Ca2+ without affecting intracellular Ca2+ levels. However, they often also have PDE3 inhibitory effects. (3) downstream mechanism: medications acting through this mechanism directly affect the connection between myosin and actin, without increasing intracellular Ca2+ levels. Due to the latter, molecules belonging to this group are not subject to the detrimental effects listed in the upstream mechanism. OM is the first drug with this mechanism of action. Notably, the terms “upstream,” “central,” and “downstream”, refer to the points in the cardiac contractile process where these drugs exert their effects.

For the second idea, as myocardial contractility constitutes a pivotal factor in the progression of heart failure, historically, drugs were constantly sought to enhance contraction at a given ventricular volume. In summary, the second mechanism is intended to accrescent myocardial contractility through any of the below 3 major categories, as shown in Figure 1.

#### 3.2.1 Upstream, by elevating the intracellular Ca2+ levels

These signaling pathways are the so-called upstream pathways of the muscle contraction process, leading to Ca2+ mobilization to raise the intracellular Ca2+ level. The classical examples can further fall into 3 subgroups, the inhibitors of Na+/Ca2+ exchanger, the activators of β1 receptors, and the phosphodiesterase 3 (PDE3) inhibitor.

Inhibitors of the Na+/K+ pump, such as Digitalis, decreases the Na+ gradient across the membrane, thus suppressing the Na+-Ca2+ exchanger, maintaining higher intracellular levels of Ca2+, consequently leading to increased contractility of cardiac myocytes (21).

The activators of β1 receptors are almost equivalent to Catecholamines. One typical instance of this class is Dobutamine which can activate the G-protein-coupled receptors that can stimulate adenylyl cyclase to transform ATP into cAMP. Therefore, when it is present, the intracellular cAMP level will be lifted. Then, the increased cAMP level will signal the protein kinase A (PKA) activation of multiple downstream receptors and pathways including the ryanodine receptors to enhance the release of Ca2+, the phospholamban to increase the Ca2+ uptake by SR through SERCA, etc., and eventually triggering a strengthened cardiac contraction (22).

As for the PDE3 inhibitors, they prevent the degradation of cAMP by PDE3, thereby activating the PKA which activates the same downstream Ca2+ cascade as catecholamines, finally achieving stronger contraction, too (23). Milrinone may serve as a representative drug in this class (24).

To sum up, any of the aforementioned drugs may lead to Ca2+ overload on cardiomyocytes, thereby raising the oxygen demand of the myocardium. Therefore, it is thought that they are energetically unfavorable due to their impact on pathways reliant on ATP (25). Besides, these drugs are likely to accelerate the heart rate, ultimately directing patients toward severe arrhythmia (26). From this aspect, OM is indeed much superior to these drugs for its Ca2+ level-independent effects in cardiac contractile enhancement. This was proven in in-vitro experiments by Ráduly et al. in cardiomyocytes isolated from dogs with entire cell membranes (27). Upon their experiments, regardless of the distinct difference in intracellular Ca2+ concentrations at systolic and resting states, OM remained intensively functional in strengthening cardiac contractility.

#### 3.2.2 In the middle, by enhancing the sensitivity to Ca2+ of myocardial cells

This is achieved through the upregulation of Troponin C which initiates muscle contraction through responses to Ca2+ binding, essentially serving as a bridge in the middle of the muscle contraction process (28). This class of drugs is relatively new, including Pimobendan, Levosimendan, etc.

However, drugs within this class are not pure Ca2+ sensitizers. The pharmacology of Pimobendan was first proposed by Fitton and Brodgen who described that its inotropic effects were derived from both the increased Ca2+ responsiveness and the PDE3 inhibition (29). Besides, Levosimendan also exhibited PDE3 inhibitory properties when it was at a relatively high plasma concentration (30). Therefore, principally, such drugs may encounter same issues as those function at upstream.

Notably, OM, the drug that was recognized as a core member of the downstream drug, was also recently found to demonstrate Ca2+ sensitizing ability under in-vitro circumstances, which will be further discussed in the following section (31).

#### 3.2.3 Downstream, by directly participating in the interaction between myosin and actin

This class of drugs is largely represented by so-called myosin activators, which can be conceptually confusing because the term is overly simplistic and, in some ways, misleading, as described by Day et al. (32).

A good example, and indeed a pioneer in terms of being the first compound discovered within this special category, is OM. OM exerts its inotropic effects through what is commonly referred to as a downstream mechanism of the muscle contraction process, influencing the mechanochemical cycle of myosin. As such, the intracellular accumulation of Ca2+ is circumvented, and thus no resulting troubles as the aforementioned drugs in abnormal energy consumption and speed-up heart rate.

The exact mechanism of action of OM, however, is sophisticated and remains not fully understood. Some believe it is a myosin activator, while others argue that it is a counterintuitive myosin inhibitor. Paradoxical results were obtained from laboratory environments and clinical trials, which will be discussed in the upcoming section.

Nevertheless, in summary, regardless of the diverse functioning mechanisms, positive inotropic agents in the big picture are thought of as emerging approaches for the treatment of heart failure in academia. However, unfortunately, due to the lack of evidence of survival benefits and concerns about the increased adverse events and even the mortality rates, routine application during the management of acute decompensated heart failure in inpatient treatment is subject to limitations (33). These drugs are primarily under consideration for specific patients who exhibit a dependency on intravenous therapy throughout their hospitalization and may serve as drug candidates for chronic outpatient positive inotropic infusion therapy. This strategy is commonly employed for patients awaiting mechanical circulatory support or heart transplantation.

## 4 The history of the discovery of OM

In 2010, Morgan et al. went alongside computer-aid design and high-throughput screening, to search for a small molecule with a specific affinity for β-cardiac myosin that was expected to be capable of being involved in the interplay between myosin, actin, ADP, Pi, and perhaps ATP as well as ATPase (34). This search ended with the discovery of CK-1827452, now known as OM. OM, the compound that was eventually synthesized, in their description, was found to increase the ratio of the time in which myosin bound to actin to that of the whole mechanochemical cycle, correspondingly, leading to greater force generation.

Later on, Malik et al. conducted their work on the dog models, unraveling its negative effects on the heart rate, peripheral vascular resistance, MAP, and ventricular end-diastolic pressure on the left heart (35). It was also observed that OM significantly increased the systolic ejection time and myocardial cell shortening fraction while avoiding the elevation of PAdP/dtmax (i.e., adjusted maximal change in pressure over time) in the left ventricle, myocardial oxygen consumption of the whole heart, and intracellular Ca2+ concentration of the myocardial cells. From that point, the efficacy of OM in the treatment of heart failure was preliminarily confirmed in the laboratory environment.

## 5 Insights into the underlying molecular mechanisms of Om

Although the fact that OM improved contractile performance without altering calcium homeostasis and metabolic consumption of myocardium and some of the preliminary understanding behind such phenomenon had been unraveled, the underlying molecular mechanisms remained largely unknown. Hence, studies concentrating on more in-depth molecular interaction have been implanted since the very first report on OM. Here, we will start from the pharmacokinetics of OM to the debates of its mechanisms of action, attempting to unravel the secret behind.

### 5.1 The metabolism of OM

The metabolism of OM has been much discussed in early phase I clinical studies, but in the past few years, attention from all walks of life has mainly focused on the macroscopic clinical effects and microscopic molecular mechanisms. Nevertheless, general understanding on OM's metabolism and basic parameters such as therapeutic concentration in human plasma were established on the basis of these work, and well summarized by Liu et al. and Trivedi et al. (36, 37). Thanks to their extraordinary contribution, we can shortly describe such backgrounds here.

In a nutshell, first above all, these early studies explored the minimal plasma level (1,200 ng/ml) in which patients might encounter dose limiting myocardial ischemic signs and symptoms, as well as the seemingly effective concentration (200–1,000 ng/ml), collectively making the practical application of OM possible (38, 39).

Moreover, as an excellent candidate drug for oral administration purpose, it was found that OM's degradation is so rarely affected by first-pass effect that around 93% bioavailability rate could be reached. Afterward, the metabolic pathways responsible for OM metabolism include the M3 biotransformation pathway (47%), CYP2D6 (27%), the inhibitory effect of ketoconazole (33%), and potentially CYP3A through the M2 pathway.

Of note that despite liver and kidney are the major characters in the play, the pharmacokinetics of OM are not significantly affected by renal function or hemodialysis, and mild to moderate liver impairment does not impact its established dosing regimen (40, 41).

### 5.2 A brief overview of the mechanochemical cycle of myosin

**Figure 2:**
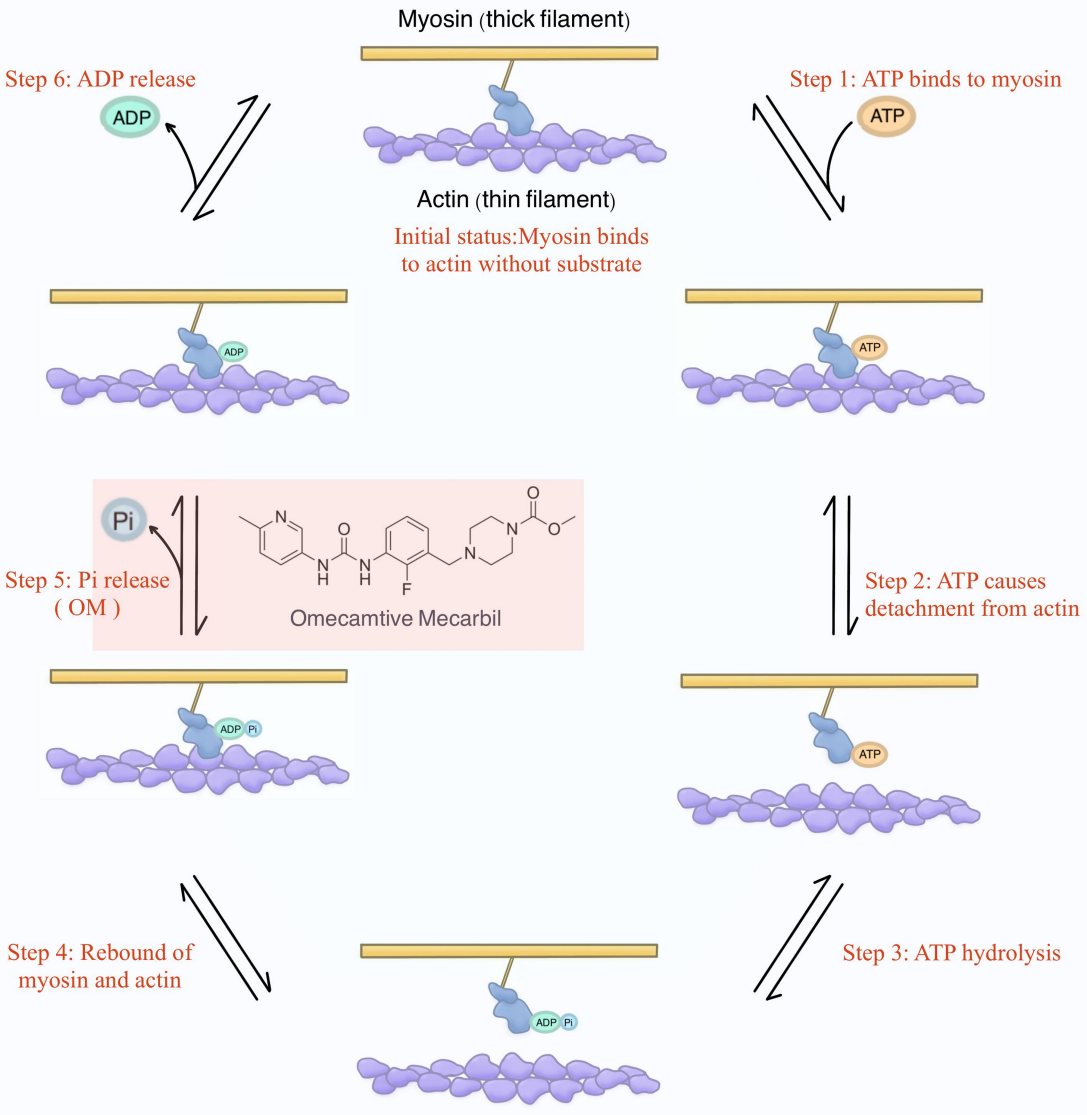
The mechanochemical cycle of myosin. Initially, envision myosin without binding to any substrates (i.e., ATP, ADP, and Pi). At this stage, myosin attaches to actin. Shortly thereafter, ATP binds to myosin, causing myosin to detach from actin. Subsequently, ATP is hydrolyzed into ADP and Pi, and they remain bound to myosin, resulting in myosin reattachment to actin. Following this, Pi is released, and the energy generated enables myosin to propel actin, initiating the relative sliding and, consequently, the development of contractile force. This step is where OM was initially proposed to exert its influence. Finally, ADP is also released, returning myosin to the initial status.

To provide a more academically refined explanation, it is imperative to delve into the intricacies of the structure and function of sarcomeres. Sarcomeres, as the quintessential contractile units responsible for generating force, exhibit nuanced variations in isoforms between cardiac and skeletal muscle fibers. Understanding the physiological underpinnings of cardiac contractility necessitates a firm grasp of knowledge of the cross-bridge formation and the relative sliding of thick filaments (i.e., myosin) and thin filaments (i.e., actin) within the sarcomere (42). This process, constituting a mechanical cycle parallelly with biochemical reactions focusing on the ATP molecule, can be principally dissected into 6 steps for precise interpretation (Figure 2).

Initial status: Commencing with myosin devoid of binding of the substrates (i.e., ATP, ADP, and Pi), the initial scenario involves myosin binding to actin.

Step 1. Subsequently, ATP engagement with myosin.
Step 2. The binding of ATP prompts the disengagement of myosin from actin.
Step 3. Sequentially, ATP hydrolysis yields ADP and Pi, which persistently remain bound to myosin.
Step 4. The hydrolysis of ATP leads to a renewed interaction between myosin and actin.
Step 5. It is at this juncture that Pi is liberated, and the ensuing energy facilitates myosin's ability to impel actin, instigating relative sliding, which is a key phase culminating in the genesis of contractile force (43). Of note, OM was initially proposed to exert its influence during this specific step in the contractile process.
Step 6. Lastly, ADP is also released, reinstating myosin to the state delineated in the initial status.

### 5.3 The Pi theory: the original understanding of the mechanism of OM

Ideally, according to the design of Morgan et al., the role of OM in the mechanochemical process of myosin is to enhance the rate of Pi release (34). This extension of the time interval during which myosin remains detached from actin after Pi release and before binding ATP leads to a larger proportion of time dedicated to this phase within the overall process. Consequently, it increases the efficiency of force generation.

This theoretical framework enjoyed continuous and consistent substantiation during the initial years. Especially in the seminal study of Malik et al., although the theory was not directly proven, OM inhibited ATPase activity, increased the apparent rate constant of phosphate release induced by actin several folds, and shifted the hydrolysis equilibrium constant towards the side in which ADP and Pi were generated (35).

Additionally, OM was found to be able to rescue the inhibition caused by R712l mutation that uncouples lever arm rotation from ATPase activity (44). This again emphasized the linkage between the underlying mechanisms of OM and ATPase activity, and hence, more or less verified the idea indirectly.

On the other hand, OM was demonstrated to significantly alter the mechanical properties of myosin, effectively augmenting the overall stall force (45, 46). From the aspect of biophysics, a mechanics study also reached similar conclusions in skinned myocardium that the acceleration of cross-bridge kinetics caused by the cardiac myosin-binding protein C (cMyBPC) ablation, the molecular basis of systolic heart failure, was largely blunted by the application of OM (47).

Overall, in the Pi theory, it was deemed that OM substantially prolonged the relative duration of myosin binding to actin during the mechanochemical cycle of myosin, bringing a matching rise in force generation between the crossbridges.

### 5.4 The inharmony voices against the original PI theory: the appearance of new theories

**Figure 3:**
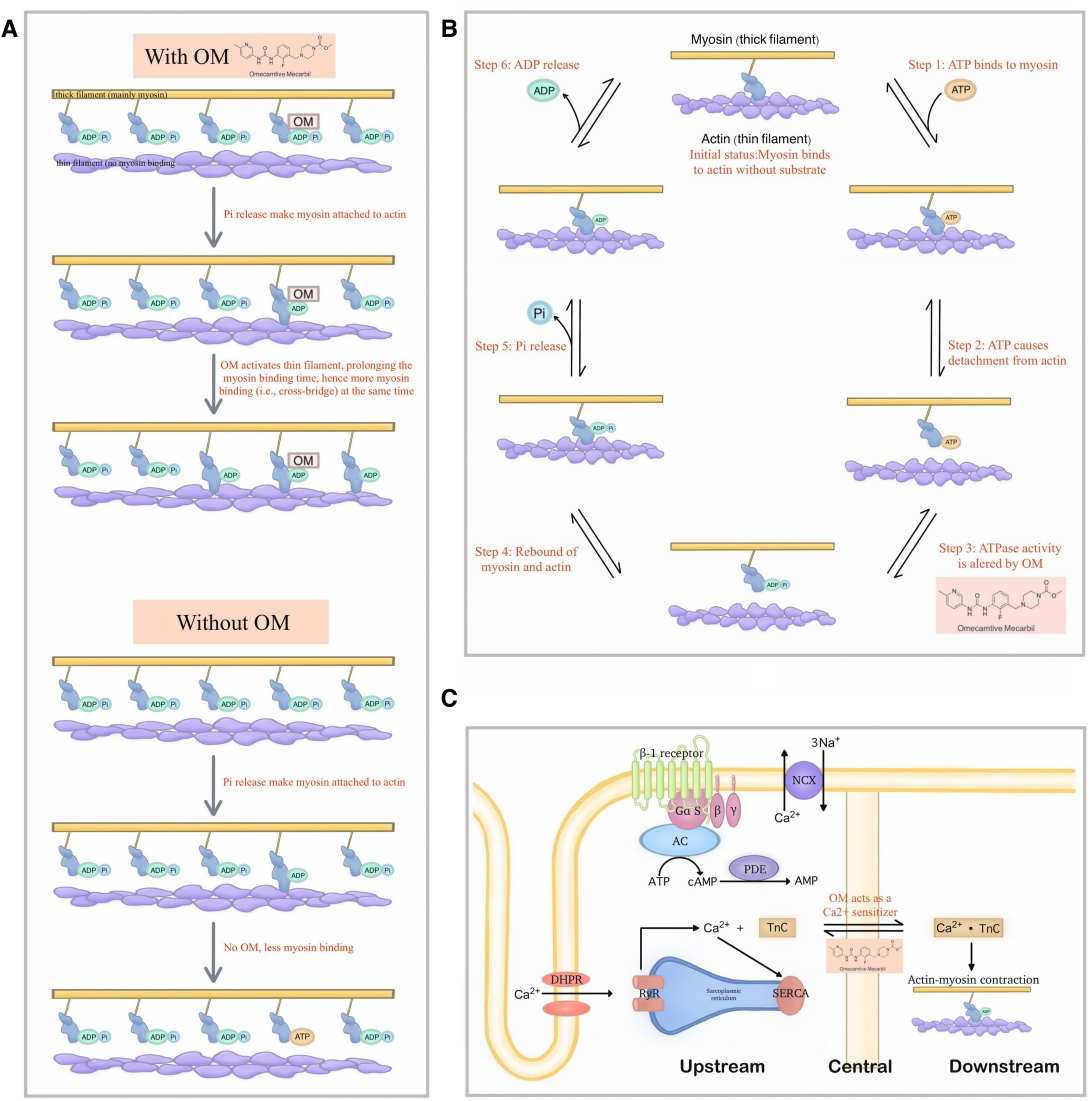
Demonstration of the new theories that explain the detailed molecular mechanism of OM. (**A**) The new model raised by Woody et al. in which OM activates the actin and thereby extends the binding time of myosin, which in turn leads to more cross-bridge formation at a given period, thereby generating greater force. (**B**) A possibility of extra effects exerted by OM on the steps of the mechanochemical cycle other than the Pi-releasing step. (**C**) OM as a Ca2+ sensitizer may also be the reason why it can demonstrate a significantly improving effect on cardiac function.

However, a turning point subsequently emerged. Several publications reported contradictory phenomena. The critical theories and the most prominent research papers in this regard are listed below (Figure 3).

#### 5.4.1 OM might be an activator of actin, instead of myosin

The *in-vitro* muscle performance assays consistently indicated that OM inhibited the relative sliding of actin across all tested concentrations (48, 49).

This was further explained by another study based on the spectroscopic analysis which suggested that OM suppressed the powerstroke by stabilizing the pre-powerstroke state of myosin (50, 51). Given that the microscopic powerstroke is thought to be positively related to the generated force in the macroscopic world, such findings confused researchers at that time.

Following that, while acknowledging the above observations, Woody et al. established the Stroke Eliminated, Prolonged Time of Attachment theory (i.e., the so-called “SEPTA model”) to account for the cardiac muscle activation in OM treatment where the observed increase in cardiomyocyte force production is due to the activation of actin, which extends the attaching time (52). The activated actin stays at the available status for more myosin combination even when it is under low Ca2+ concentration, thereby increasing the number of bound myosin at a given period, forming more cross-bridges which in turn generate greater force.

#### 5.4.2 The efficacy of OM might also come from the alteration of ATPase activity

Albeit in a non-human species, an early study suggested that OM influenced steps beyond Pi release, resulting in a reduction in the activity of the enzyme responsible for ATP hydrolysis (53). In other words, the microscopic way how OM exerted its net effects in the macroscopic world, is multifaced. Hence, it is not reasonable to be excluded the possibility that instead of solely attributing the improved cardiac contraction to the Pi release, the retardation of ATP degradation can also be the main contributor.

This concept challenges the fundamental theory where OM was said to achieve its effects by influencing the release of Pi, as it introduces an alternative possibility in the context of OM's direct interaction with myosin.

However, noteworthily, another research figured out the opposite result, proposing that OM can elevate myocardial oxygen consumption, and this phenomenon was attributed to the excessive activity of myosin ATPase (54).

#### 5.4.3 OM might also act as a Ca2+ sensitizer, promoting the performance of the heart

Interestingly, besides the theories around the ATPase activity, Nagy et al. found that OM affected the Ca2+-force relationship in permeabilized cardiomyocyte-sized preparations of rats under isometric conditions. Their results suggested that OM, in addition to the well-known direct activation effects on myosin, could also strengthen cardiac contractility by increasing the cellular sensitivity to Ca2+ (31).

Similar findings were gained by Gollapudi et al., but they thought both the Ca2+ sensitization and slower force generation were owed to the increased duty ratio caused by OM within the mechanochemical cycle of myosin, thereby bridging the original Pi theory with another fundamental mechanism of inotropic agents (55).

Although there is ongoing disagreement over the mechanism of action of OM, it is evident that OM is a small molecule that is capable of directly binding to myosin and demonstrating therapeutic effects under in-vivo conditions. Hence, there exists a widely held view that myosin might serve as a feasible target for drug development, and OM may emerge as a highly auspicious option for medicinal purposes.

## 6 The clinical trials with OM

The objective of pharmacotherapy in heart failure management is to alleviate the clinical symptoms in connection with heart failure including manifestations such as edema, dyspnea, and reduced exercise tolerance, to ameliorate the quality of life especially minimizing the chance of hospitalization, and to lower the mortality rate (3, 5, 56–58). From this aspect, Cytokinetics, Amgen, Servier, etc., the international big pharma corps, funded a series of multi-centered, double-blind, placebo-controlled, randomized clinical trials (i.e., ATOMIC-HF, COSMIC-HF, GALACTIC-HF, and METEORIC-HF) for the prospective application of OM in the treatment of heart failure. The aforementioned efforts are currently regarded publicly as the largest and most authorized data in the field of cardiology. A summary of these large-scale clinical trials was organized in the form of a table as shown below (Table 2).

**Table 2:** Summary of the large-scale clinical trials with OM at present.

Clinical trial	Results published	Trial design	Endpoints	Primary findings
ATOMIC-HF	PMID: 27012405	Phase IIb, double-blind, placebo-controlled, randomized	Primary: effect on dyspnea of 48 h of intravenous OM administration	Primary endpoint: missed
			Secondary: safety and tolerability of the 3 dose levels of OM	Secondary endpoint: met
COSMIC-HF	PMID: 27914656	Phase II, double-blind, placebo-controlled, randomized	Primary: safety, tolerability, and pharmacokinetics of the oral dosage of OM	Primary endpoint: met
			Secondary: change from baseline in systolic ejection time, stroke volume, left ventricular end-systolic and end-diastolic diameters, heart rate, and the level of NT-proBNP	Secondary endpoint: met
GALACTIC-HF	PMID: 33185990	Phase III, double-blind, placebo-controlled, randomized	Primary: time to cardiovascular death or first heart failure event	Primary endpoint: met
			Secondary: time to CV death, patient reported outcomes measured by Kansas City Cardiomyopathy Questionnaire, time to first heart failure hospitalization and time to all-cause death	Secondary endpoint: missed
METEORIC-HF	PMID: 35852527	Phase III, double-blind, placebo-controlled, randomized	Primary: change in peak oxygen uptake on cardiopulmonary exercise testing	Primary endpoint: missed
			Secondary: alterations in peak exercise workload, ventilatory efficiency, and average daily activity units	Secondary endpoint: missed

### 6.1 The ATOMIC-HF trial

The ATOMIC-HF trial is a double-blind, randomized, placebo-controlled sequential cohort phase IIb clinical trial designed to evaluate an intravenous formulation of OM in approximately 600 patients hospitalized with acutely decompensated heart failure in three sequential, ascending-dose cohorts (59–61).

The primary efficacy endpoint was centered on dyspnea in the presence of OM to see if the symptoms of dyspnea improved within 48 h without worsening heart failure or death from any cause. Secondary and exploratory endpoints were safety, tolerability, pharmacokinetics, and echocardiographic indices.

At the end of the trial, it was found that intravenous OM did not significantly improve dyspnea symptoms in hospitalized patients with acute decompensated heart failure. However, statistics show that the patient cohorts show a trend toward greater response as the OM dose increases. Furthermore, the concentration-dependent increase in systolic ejection time was found to be statistically significant, strongly suggesting an encouraging improvement in cardiac function. On the other hand, as a phase IIb clinical trial, despite the lack of primary efficacy endpoints, the safety and tolerability of OM were guaranteed, so it was still considered a great success.

### 6.2 The COSMIC-HF trial

COSMIC-HF is a phase II, multicenter, randomized, double-blind, placebo-controlled clinical trial organized by Amgen in collaboration with Cytokinetics to meticulously assess and ascertain the impact of an orally administered modified sustained-release formulation of OM within a cohort of subjects afflicted with heart failure and left ventricular systolic dysfunction (62).

In total, there were 448 patients from 13 countries enrolled in the trial and randomized to receive either placebo or treatment with OM dosed as 25 mg per day or 25 mg with dose escalation to 50 mg twice per day. The intact trial lasted for 20 weeks and participants were followed for a total of 24 weeks.

The primary endpoint of the trial was to assess the maximal OM concentration in plasma, and the secondary endpoints aimed to examine changes from baseline in various clinical parameters at week 20, including systolic ejection time, stroke volume, left ventricular end-systolic diameter, left ventricular end-diastolic diameter, heart rate, and NT-proBNP (i.e., a biomarker associated with the severity of heart failure), and the safety and tolerability of OM that were measured by the assessment of adverse events incidence from baseline to week 24.

The findings reveal that, compared with what could be observed from the placebo-controlled group, the oral administration of OM guided by a pharmacokinetic-based dosing strategy yields significant improvements in cardiac function and a reduction in ventricular dimensions, meanwhile adverse and clinical events in patients on OM were comparable to placebo meaning that the OM group sustained an equivalent safety and tolerability.

### 6.3 The GALACTIC-HF trial

Inspired by the success obtained from the phase II clinical trials (i.e., ATOMIC-HF and COSMIC-HF), a phase III clinical trial called GALACTIC-HF was initiated with 8,256 HFrEF patients from 35 countries recruited and administered with OM or placebo for a median of 21 months (63).

In the trial, patients were randomly assigned to 2 groups, one receiving the oral placebo and the other taking OM at an initial dose of 25 mg, twice daily. The primary composite study endpoint was defined as the time until the occurrence of cardiovascular death or the first onset of heart failure. Secondary endpoints encompassed the time to cardiovascular death, patient-reported outcomes measured by the Kansas City Cardiomyopathy Questionnaire, time to first heart failure hospitalization, and time to all-cause death.

In contrast to the placebo cohort, the administration of OM exhibited noteworthy success in attaining the primary composite efficacy endpoint. This intervention led to a statistically significant reduction in the incidence rate of cardiovascular mortality or heart failure events, and patients receiving OM, in general, manifested fewer symptoms, with lower NT-proBNP levels compared to patients receiving the placebo.

Significant insights have been gleaned from the extensive dataset, yielding fruitful results through secondary analyses. Subsequent analysis indicated a progressive enhancement of OM's therapeutic efficacy as the EF declined (64). Moreover, a *post hoc* analysis suggested that the use of OM in patients with severe heart failure might potentially reach a reduction in the time to the composite endpoint of the first heart failure event or cardiovascular death (65). Another *post hoc* analysis pointed out that OM presented good efficacy and tolerability in patients with HFrEF combined with low systolic blood pressure, indicating that OM might be of particular benefit for those patients (66).

However, the results were still considered disappointing from the side of investment, as previously analysts expected a way more obvious reduction in the incidence rate of cardiovascular mortality or heart failure events to make it more competitive than other new drugs under development such as mavacamten (67–71). Furthermore, no secondary endpoints were observably improved, and it did not impart an extension in the survival duration among the high-risk patient population, which no doubt worsened the situation.

### 6.4 The METEORIC-HF trial

As exercise intolerance is a predominant clinical symptom in patients with HFrEF, and current pharmacological interventions recommended in the guidelines do not consistently yield sustained improvements in patients' exercise tolerance, most recently, another phase III trial, METEORIC-HF, was set up to investigate if OM could contribute to addressing the present issue (72).

In the trial, patients with heart failure were selected based on specific criteria, including a left ventricular EF of less than or equal to 35%, NYHA functional classification class II to III, NT-proBNP concentration greater than or equal to 200 pg/ml, and a peak VO2 less than or equal to 75%. These patients were subjected to a random allocation in a 2:1 ratio into 2 groups, the OM group, and the placebo-controlled group. The OM group received varying dosages of the drug during a 20-week-long treatment, with subsequent follow-ups. The primary endpoint of the trial focused on assessing changes in peak VO2, while secondary endpoints encompassed evaluating alterations in peak exercise workload, ventilatory efficiency, and average daily activity units.

Regretfully, as a result of this exploration, the OM-treated group exhibited no statistically significant alterations in peak VO2, maximal exercise workload, and ventilatory efficiency in comparison to the placebo-controlled group. This implied that OM was not likely to be a solution for exercise tolerance for HFrEF patients, at least a 20-week-like short-term administration would not be. Together with the less satisfying performance of OM in the GALACTIC-HF trial, this may potentially exacerbate the challenges in the commercialization process of OM.

## 7 Beyond the failing heart: the comorbidities consideration of OM administration

It is widely recognized that the occurrence of acute or chronic dysfunction in one organ frequently contributes to the exacerbation of acute or chronic dysfunction in another organ, and the heart is not an exception to this phenomenon. Heart failure is associated with a substantial prevalence of comorbidities, which can augment the likelihood of death and diminish the overall quality of life. Hence, the presence of comorbidities significantly influences both the clinical manifestation and long-term outlook of individuals with heart failure (73, 74). Complications that accompany heart failure, including chronic renal impairment, chronic pulmonary diseases, osteoarthritis, etc., may warrant supplementary therapeutics.

Currently, in addition to the main findings, further data analysis has revealed that OM also has potential in the prevention of concurrent disorders commonly linked to heart failure. An illustrative instance may be observed in a *post hoc* analysis of the previously described clinical trials, in which chronic kidney disease (CKD), one of the most prevalent noncardiac comorbidities, was found to significantly influence clinical symptoms and treatment outcomes and might be of potential abatement by OM application (75–78). In the study of Beldhuis et al., the renal complications accompanying heart failure were eased (79). In light of this, for patients with advanced stage 4 CKD, the utilization of OM could be of interest. The recommendation is supported by the inclusion of patients with an estimated glomerular filtration rate of 20 ml/min/1.73 m^2^ or higher in the GALACTIC-HF trial.

## 8 Concerns about the use of OM

**Figure 4:**
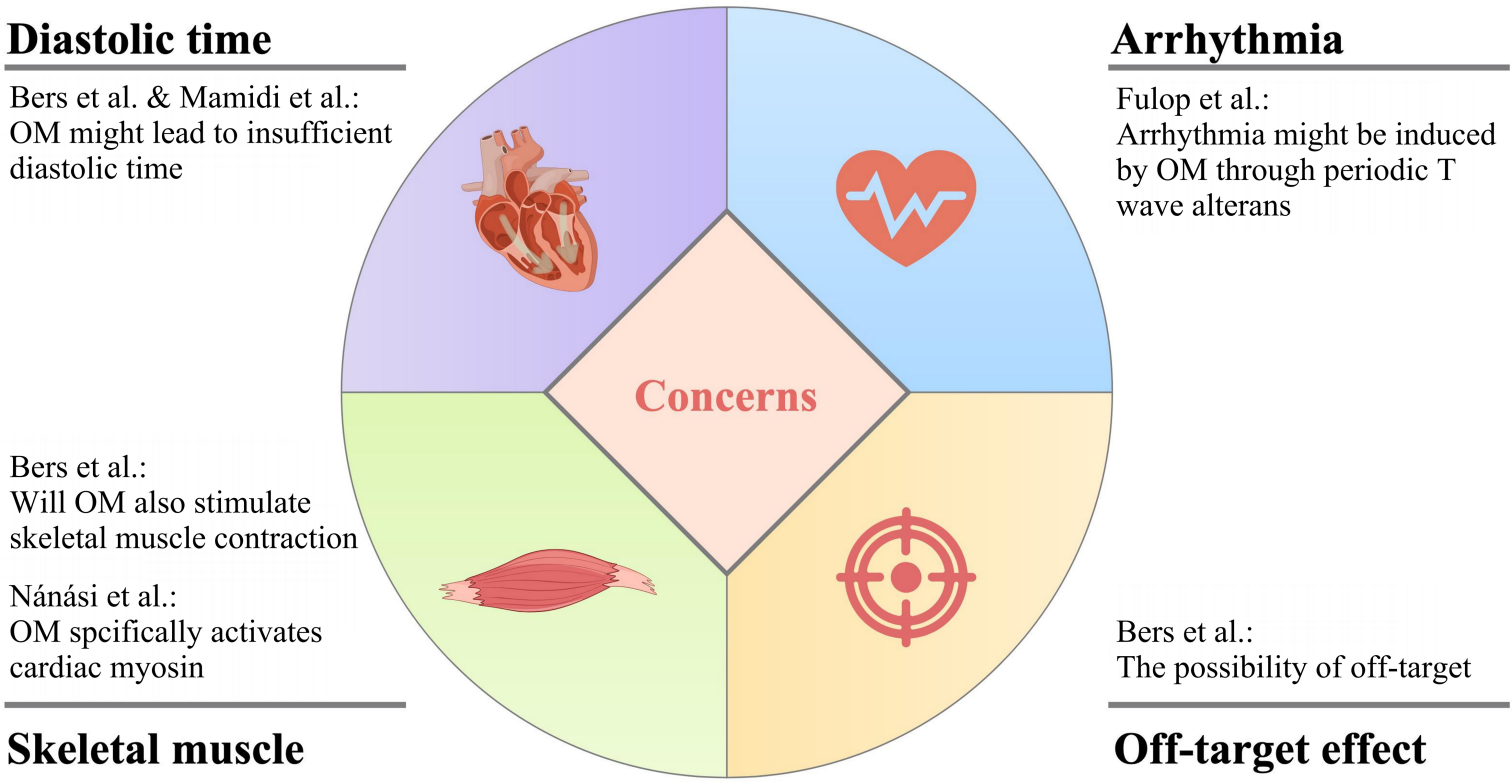
The main concerns of OM application.

While OM exhibits promising attributes for the treatment of heart failure, it is imperative to acknowledge that there exist certain pertinent concerns that warrant careful consideration and thorough investigation to comprehensively assess its viability and safety as a therapeutic intervention for this complex medical condition. The main concerns of OM application are summarized in Figure 4.

### 8.1 Will OM cause insufficient diastolic time?

The administration of OM elicits a cardiac response characterized by an increase in systolic ejection time, which is considered a hallmark of myosin activation. However, this alteration is concomitant with a proportional reduction in diastolic duration at a specific heart rate. This hypothesis is not unfounded, as demonstrated in the research done by Liu et al., OM induced a higher proportion of firmly attached cross-bridges in the total amount of cross-bridges that could be possibly formed than when OM was absent, leading to a decrease in the in-vitro motility velocity which suggested that the drug was likely to result in slower cardiac contraction (53). Because of the prolongation of systolic duration caused by OM, there is a probability for the patient to experience inadequate diastolic time, and ultimately insufficient heart reperfusion (26, 80).

In fact, later, Fülöp et al. found that under clinical concentration, in the presence of OM, the cardiac output was not increased, but the EF was higher due to the decreased diastolic volume in the left ventricle in rat models (81). This, with no doubt, further suggested that OM might have negative effects on the diastolic function of the heart, pushing the issue back to the table.

### 8.2 Is there a risk of arrhythmia due to the periodic electromechanical alternans?

The work of Fülöp et al., in their own words, featured two novel points, namely, the aforementioned diastolic problems, and the dose- and heart rate-dependent, transient, as well as pulsus alternans-like electromechanical alterations (81). Graphically, this change was shown as T wave alternans in the ECG, with no modification in QRS morphology. Interestingly, in the past literature, there were also opinions linking the alternative T wave pattern to the onset of a life-threatening arrhythmia, which to a certain extent cross-validated the results of Fülöp et al. (82).

It is not unreasonable that OM is suspected of causing arrhythmias in patients with heart failure. Before the investigation of Nagy et al. in which OM was demonstrated to be a Ca2+ sensitizer, accumulating evidence suggested that Ca2+ sensitization was positively related to the occurrence of tachyarrhythmias in the left ventricle (31, 83–85).

Taking it all together, OM likely potentiates arrhythmia, and here comes a new question: the two issues involved in subsections 7.1 and 7.2 alone are enough to make people reconsider whether the improvement in cardiac systolic function caused by OM can be offset by diastolic dysfunction and arrhythmias also caused by OM. Therefore, there is a long way to go to clarify the clinical benefits that heart failure patients can achieve after using OM.

### 8.3 Will OM also stimulate skeletal muscle contraction?

The direct activation of myosin by OM enhances the generation of force in cardiac muscle cells, thereby salvaging the cardiovascular system. However, it is worth being aware that this mechanism might also impact the functionality of other systems.

For instance, since slow skeletal muscle fibers commonly express an isoform of cardiac myosin heavy chain, the Ca2+ sensitization effect of OM has been observed not only in cardiac muscle cells but also in skeletal muscle fibers of the diaphragm in rats. Consequently, it implies that various physiological activities involving skeletal muscles may potentially be impaired following OM administration (12, 53).

From an indirect aspect, a piece of good news was supporting the clinical application of OM against the above concern, in which OM activated the ryanodine receptors from cardiomyocytes with relatively high specificity instead of skeletal muscle cells (86).

Besides, of note an interesting fact that while OM suppressed the powerstroke generated by normal cardiomyocytes, it was found able to rescue R712l-myosin working stroke, aβ-cardiac myosin motor mutation that could lead to a severe hypertrophic cardiomyopathy (44).

### 8.4 The possibility of off-target

The term “off-target” pertains to adverse effects arising from the modulation of additional targets, which can be either biologically related or entirely unrelated to the primary target of interest. It is common knowledge that when a drug is off-target, toxicity to the human body can be aroused. This is the so-called “side effect”.

Similarly, when OM interacts with off-targets, it may potentially exert deleterious effects on the human body, exacerbating the patient's condition. Therefore, further in-depth research into the stability of OM's target is warranted.

In a previous study, it was reported that although OM exerted its effects on the Ca2+ transient in a concentration-dependent manner, in case its concentration exceeded a certain limit, the off-target effect might occur, and it mainly functioned through such mechanisms (87).

### 8.5 Drug-drug interaction

Since 2021, Trivedi et al. have been working on identifying the potential drug-drug interaction of OM form regularly used drugs for heart failure. So far, they had exanimated the co-administration of OM and CYP3A4 substrates, digoxin, amiodarone, and omeprazole (88–90). For rosuvastatin, with OM, its systemic exposure was slightly increased, but overall, the combinatory application was considered to be safe and well-tolerated (91).

### 8.6 Opponent reports

While the majority of the scientific community members are expecting such promising drug come into real practice, there remain some reports claiming that no positive inotropic effect of OM either in human atrium or human ventricle could be observed after administrating OM.

To be more specific, in the work of Dashwood et al., they found that in a failing human heart, while OM preserving contractile force and thus stopping further collapse, it has negative effects on diastole. Such adverse effects needed to be rescued by (-)-noradrenaline (92). In the study of Lina et al., their experiment demonstrated complying results as Dashwood et al., and further concluded that OM was rather a negative inotropic agent instead of a positive one (93).

## 9 Discussion

Heart failure represents a multifaceted clinical syndrome, distinguished by its ubiquity, substantial financial burden, debilitative consequences, and potential for fatal outcomes. It arises from the heart's incapacity to adequately supply the necessary blood and oxygen to meet the body's metabolic demands (6). The prevalence of this condition within the general populace spans a range of 0.3% to 2%, with an even higher proportion of 6% to 10% among individuals aged 65 and above. The rising prevalence of heart failure can be attributed to a variety of factors, including aging and the increased prevalence of risk factors such as diabetes, obesity, hypertension, and atherosclerosis (94). The increasing patient population emphasizes the importance of effective heart failure prevention and management as a global public health challenge (1–3).

Despite notable progress in treatment strategies, the prognosis remains less favorable. Alongside technical considerations, economic factors frequently substantially influence clinical outcomes. As Bers et al. have underscored, cardiac transplantation remains the sole definitive recourse for heart failure, albeit a significant proportion of patients opt against this therapeutic option due to its considerable financial burdens (26).

**Figure 5:**
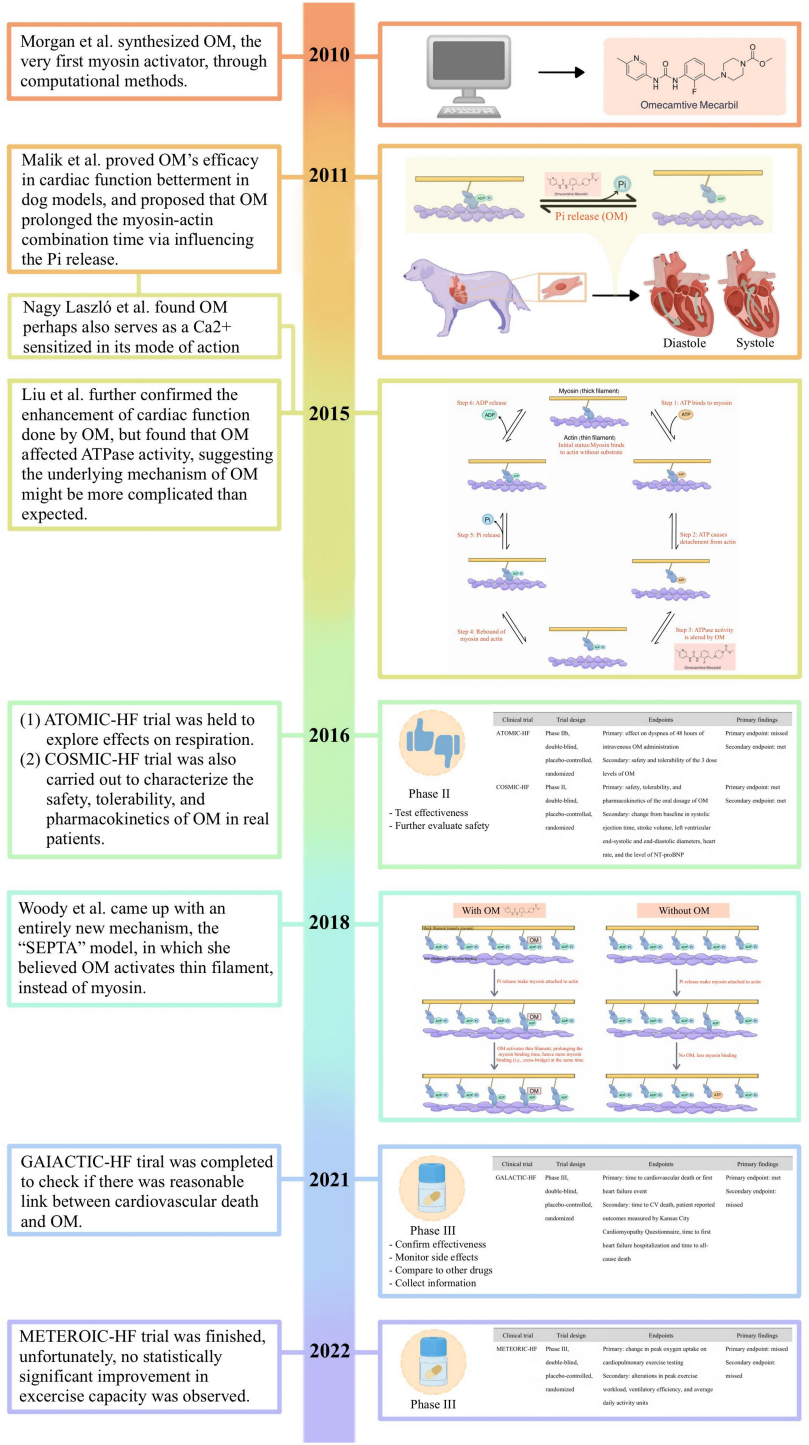
The key historical points of its development following the timeline.

Therefore, pharmacological therapies, which are comparatively less costly, serve as an essential component in the management of this pervasive condition. Within this context, it is irrefutable that emerging pharmaceutical agents, exemplified by OM, which hold the potential to augment cardiac function, furnish a crucial lifeline for afflicted patients. While still in the nascent stages of development, the research outlined above suggested that modulating cardiac function through myosin activators represents a promising therapeutic strategy for an array of myocardial diseases and heart failure, thus holding vast prospects for the field (95, 96). Herein, we demonstrated the key historical points of its development following the timeline (Figure 5).

All in all, OM is thought to be a candidate agent in the treatment of heart failure with huge potential. It principally targets the force generation process by the mechanochemical cycle of myosin either directly activating myosin or actin, successfully avoiding the unwanted effects raised by the intervention on neuro-humoral systems or by the upstream Ca2+ signaling, while possessing an excellent performance in the betterment of the contractility of the myocardium. Although the exact mechanisms behind OM's magic in the failing heart remain under debate, its outstanding amelioration in cardiac contractility has been broadly accepted by the medical domain.

Clinically, its efficacy and safety were verified, too, although certain expectations such as exercise capability were not reached. Besides, some comorbidities including chronic renal disease have proven to be beneficial from the application of OM.

The oral administration capability of OM distinguishes it from other intravenous therapies targeting contractility enhancement, which makes its use more practical in a patient's daily life.

Besides, as numerous publications have highlighted the efficacy of OM appears to vary significantly across distinct subgroups of heart failure patients. Therefore, a precise patient selection process that identifies those likely to derive benefits from this medication is anticipated to be refined in the foreseeable future.

However, prior to the deployment of OM on a large scale, as indicated by other researchers, some concerns need to be addressed, including whether sufficient diastolic time can be ensured, if any arrhythmia will be induced, the unexpected effects on the other systems such as skeletal muscle, and the existence of off-target effect (26, 81, 87).

In addition, there is room for improvement in some specific clinical use issues. Although not extensively discussed in the present article due to the lack of available original studies, especially those in human ones, it is worth thinking of the optimal dose of the drug that should be applied to patients. This is because previous studies in canine cardiomyocytes showed that once OM was under excessive concentration, it might decrease, rather than increase the cardiac contractility, highlighting that a more cautious dose of OM used in reality should be clarified (87, 97).

## 10 Summary

The present article summarized the latest research in relevance to Omecamtiv Mecarbil in the treatment of heart failure.

More specifically, the review first elucidated the pathophysiological basis of heart failure, its classification, and the current strategies in developing pharmaceutic agents against it. Then, the underlying mechanisms of Omecamtiv Mecarbil were discussed, together with the history of how it was synthesized. Lastly, clinical trials and relevant concerns were also introduced and demonstrated, followed by a rational prospect.

In a nutshell, although some obstacles remain, the modulation of heart function through myosin activators, especially Omecamtiv Mecarbil, is potentially paving the way for future therapies that directly interact with contractile proteins to enhance myocardial force.
